# Diallyl Trisulfide Protects Rat Brain Tissue against the Damage Induced by Ischemia-Reperfusion through the Nrf2 Pathway

**DOI:** 10.3390/antiox8090410

**Published:** 2019-09-18

**Authors:** Carlos A. Silva-Islas, María E. Chánez-Cárdenas, Diana Barrera-Oviedo, Alma Ortiz-Plata, José Pedraza-Chaverri, Perla D. Maldonado

**Affiliations:** 1 Laboratorio de Patología Vascular Cerebral, Instituto Nacional de Neurología y Neurocirugía, CDMX 14269, Mexico; charlssilv@gmail.com (C.A.S.-I.); echanezc@gmail.com (M.E.C.-C.); 2 Departamento de Farmacología, Facultad de Medicina, Universidad Nacional Autónoma de México, CDMX 04510, Mexico; dianabarrera@hotmail.com; 3 Laboratorio de Patología Experimental, Instituto Nacional de Neurología y Neurocirugía, CDMX 14269, Mexico; aortizplata@yahoo.com.mx; 4 Departamento de Biología, Facultad de Química, Universidad Nacional Autónoma de México, CDMX 04510, Mexico; pedraza@unam.mx

**Keywords:** cerebral ischemia, diallyl trisulfide, Nrf2, stroke, brain, ischemia-reperfusion, SOD1, SOD2, GST

## Abstract

Stroke is a public health problem due to its high mortality and disability rates; despite these, the pharmacological treatments are limited. Oxidative stress plays an important role in cerebral damage in stroke and the activation of the nuclear factor erythroid 2-related factor 2 (Nrf2) confers protection against oxidative stress. Different compounds, such as diallyl trisulfide (DATS), have the ability to activate Nrf2. DATS protects against the damage induced in oxygen-glucose deprivation in neuronal cells; however, in in vivo models of cerebral ischemia, DATS has not been evaluated. Male Wistar rats were subjected to 1 h of ischemia and seven days of reperfusion and the protective effect of DATS was evaluated. DATS administration (IR + DATS) decreased the infarct area and brain damage in the striatum and cortex; improved neurological function; decreased malondialdehyde and metalloproteinase-9 levels; increased Nrf2 activation in the cortex and the expression of superoxide dismutase 1 (SOD1) in the nucleus, SOD2 and glutathione S-transferase (GST) in the striatum and cortex; and increased the activity of catalase (CAT) in the striatum and glutathione peroxidase (GPx) in the cortex. Our results demonstrate the protective effect of DATS in an in vivo model of cerebral ischemia that involves Nrf2 activation.

## 1. Introduction

Stroke is a cerebral circulatory disorder wherein one or more blood vessels are affected, altering temporarily or permanently the function of the brain [1,2]. Stroke is a public health problem because in 2015, it represented the second cause of death worldwide and was the leading cause of adult disability in industrialized countries [3]. It also represents an economic challenge, since 15% to 30% of the survivors become permanently disabled and 20% require institutional care Reviewed in [4]. Cerebral ischemia represents 87% of stroke cases [3] and occurs by the permanent or transitory occlusion of cerebral blood vessels, caused primarily by a thrombus or embolus that decreases or blocks the cerebral blood flow [2,4]. The transient occlusion of cerebral blood flow induces a series of biochemical events known as ischemic cascade, which include: (1) depletion of ATP levels; (2) anaerobic glycolysis; (3) lactic acidosis; (4) membrane depolarization; (5) excitotoxicity; (6) mitochondrial dysfunction; (7) reactive oxygen species (ROS) and reactive nitrogen species (RNS) production; (8) oxidative stress; and (9) inflammation, leading to neuronal death Reviewed in [4,5].

There are reports indicating that ROS and RNS are produced during reperfusion, as a consequence of ATP depletion, mitochondrial dysfunction, increase in intracellular Ca^2+^ levels and the restoration of the oxygen and glucose supply [6,7]. The increase in ROS and RNS induces the oxidation of lipids, proteins and deoxyribonucleic acid (DNA), leading to cellular integrity and function impairment, eventually causing neuronal death [7]. The nuclear factor erythroid 2-related factor 2 (Nrf2) is the master regulator of the oxidative stress response due to its ability to induce the transcription of antioxidant and phase 2 proteins, such as heme oxygenase-1 (HO-1), NAD(P)H quinone oxidoreductase (NQO1), superoxide dismutase 1 (SOD1), SOD2, glutathione S-transferase (GST), glutathione peroxidase (GPx), glutathione reductase (GR) and catalase (CAT), which together are capable to regulate the cellular redox state by decreasing ROS levels [8,9]. In homeostatic redox conditions, Nrf2 is sequestered in the cytoplasm by the Kelch-like ECH-associated protein 1 (Keap1) [10], a substrate adaptor protein for Cullin3-based Cullin-RING E3 ubiquitin ligase [11], which binds to the ETGE and DLG motifs in Nrf2 through its Kelch domain [12], promoting Nrf2 ubiquitination and degradation by 26S proteasome [11]. Conversely, in the presence of free radicals or electrophilic compounds such as tert-butylhydroquinone (tBHQ) or sulforaphane (SULF), some cysteines in Keap1 are oxidized, promoting Keap1-Nrf2 interaction disruption, decreasing Nrf2 degradation and increasing its nuclear translocation [13]. In the nucleus, Nrf2 is dimerized with small Maf proteins (MafK, MafG, MafF) and binds to antioxidant response elements (ARE), inducing the transcription of a variety of protective genes [14].

Garlic is a member of the Amaryllidaceae family that exhibits different biological activities such as antioxidant, antitumoral, anti-atherosclerosis, anti-inflammatory, and detoxifying properties [15,16,17,18,19,20]. Garlic oil, a garlic presentation, is composed of different organosulfur compounds such as diallyl sulfide (DAS), diallyl disulfide (DADS) and diallyl trisulfide (DATS), which also exhibit some protective cellular properties. DATS is one of the major components in garlic oil [19]; it exhibits anticoagulant properties [21], antioxidant activity [19,22,23,24] and protects against the damage induced by amyotrophic lateral sclerosis [25] and myocardial ischemia [26]. DATS shows direct antioxidant capacity since it is capable to scavenge free radicals such as superoxide anion radical (O_2_^.−^) and peroxynitrite (ONOO^−^) [22]; and indirect antioxidant capacity because is able to activate the Nrf2 pathway [27]. There are reports indicating the ability of DATS to induce phase 2 enzymes such as GST, GPx, GR [23,24], HO-1 [25,28] and NQO1 in a Nrf2-dependent way [18]; DATS is also capable of increasing glutathione (GSH) levels [24]. These properties of DATS to activate the Nrf2 pathway led us to use this compound derived from garlic oil in an in vivo model of ischemic stroke in rats as a possible neuroprotective agent through the activation of the Nrf2 signaling pathway.

## 2. Materials and Methods 

### 2.1. Reagents

DATS was obtained from LKT laboratories (St. Paul, MN, USA). Folin, GSH, hydrogen peroxide (H_2_O_2_), dithiothreitol (DTT), phenylmethylsulfonyl fluoride (PMSF), paraformaldehyde (PFA), β-nicotinamide adenine dinucleotide phosphate reduced (NADPH), GR, pepstatin A, aprotinin, leupeptin, Nonidet^®^ P40, Triton^®^ × 100, ethylenediaminetetraacetic acid (EDTA), phosphatases cocktail inhibitor and bovine serum albumin (BSA) were obtained from Sigma (St. Louis, MO, USA). Sodium azide (NaN_3_) was obtained from Hycel de México (Zapopan, Jalisco, México). Hydroxyethyl piperazineethanesulfonic acid (HEPES) was from MP Biomedicals (Solon, OH, USA). Primary antibodies rabbit anti-GST (110-218; sc-33614) and rabbit anti-matrix metalloproteinase-9 (MMP-9; H-129; sc-10737) were obtained from Santa Cruz Biotechnology (Dallas, TX, USA). The primary antibody rabbit anti-malondialdehyde (MDA; ab6463) was obtained from Abcam (Cambridge, MA, USA). Primary antibodies rabbit anti-SOD1 (ADI-SOD-101-E) and rabbit anti-SOD2 (ADI-SOD-111) were purchased from Enzo Life Science (Farmingdale, NY, USA). TransAM Nrf2 ELISA kit was from Active Motif (Carlsbad, CA, USA) and Universal L Kit SAB-System HRP was from DAKO (Carpinteria, CA, USA). All other reagents were obtained from other known commercial local sources.

### 2.2. Animals

Male Wistar rats (280–320 g), three months old, were housed under controlled conditions of light (12 h light/dark cycles) and temperature (25 °C ± 3 °C) in acrylic boxes (4 rats per cage) with food (Laboratory rodent diet 5001; PMI Feeds Inc., Richmond, IN, USA) and water ad libitum. All procedures with animals were carried out strictly according to the National Institutes of Health for the Care and Use of Laboratory Animals and the Local Guidelines on the Ethical Use of Animals from the Health Ministry of Mexico (NOM-062-ZOO-1999 SAGARPA) and were approved by the Local Ethics Committee of the Instituto Nacional de Neurología y Neurocirugía (INNN 117/09 project approved on 10 May 2010). All efforts were made to optimize the number of animals used and minimize their suffering.

### 2.3. Experimental Design

Animals were randomly divided into four groups (*n* = 3–6) as follows: (1) control group (CT); (2) diallyl trisulfide group (DATS); (3) ischemia/reperfusion group (IR); and (4) IR plus DATS group (IR + DATS). Animals from IR and IR + DATS groups were subjected to 1 h of ischemia and 7 days of reperfusion as detailed below. Animals from DATS and IR + DATS groups received 4 doses of DATS (15 mg/Kg, i.p.) every 24 h. The first dose of DATS in the IR + DATS group was administered 5 min before the onset of reperfusion and the amount of DATS corresponding to each rat was dissolved in 200 µL of corn oil.

Animals were sacrificed 7 days after the onset of reperfusion or the first dose of DATS. The brains were removed, dehydrated and paraffin embedded. Coronal sections were done for the histology and immunohistochemistry assays, as detailed below. Furthermore, for Nrf2 activation assay, animals were sacrificed and the brain cortex was obtained and homogenized immediately, as explained in the nuclear extraction section. Finally, animals used for enzyme activity were sacrificed and the striatal and cortex tissues were obtained and stored immediately at −70 °C until use. 

### 2.4. Middle Cerebral Artery Occlusion (MCAO) Model 

The MCAO was made as previously reported by Longa et al. [29]. Animals were anesthetized with a mixture of isoflurane (2.5%), oxygen (2%) and nitrogen (80%) and an incision of 1 cm was performed in the suprasternal region. The carotid artery was dissected followed by the insertion of a 20 mm filament of 3–0 nylon in the external carotid, passing through the common carotid artery and the internal carotid to block the middle cerebral artery. Animals were closed to allow recovery and after 1 h of the MCAO, animals were anesthetized and the filament was removed to restore the blood flow, thus starting the reperfusion. 

### 2.5. Histology

**Figure 1:**
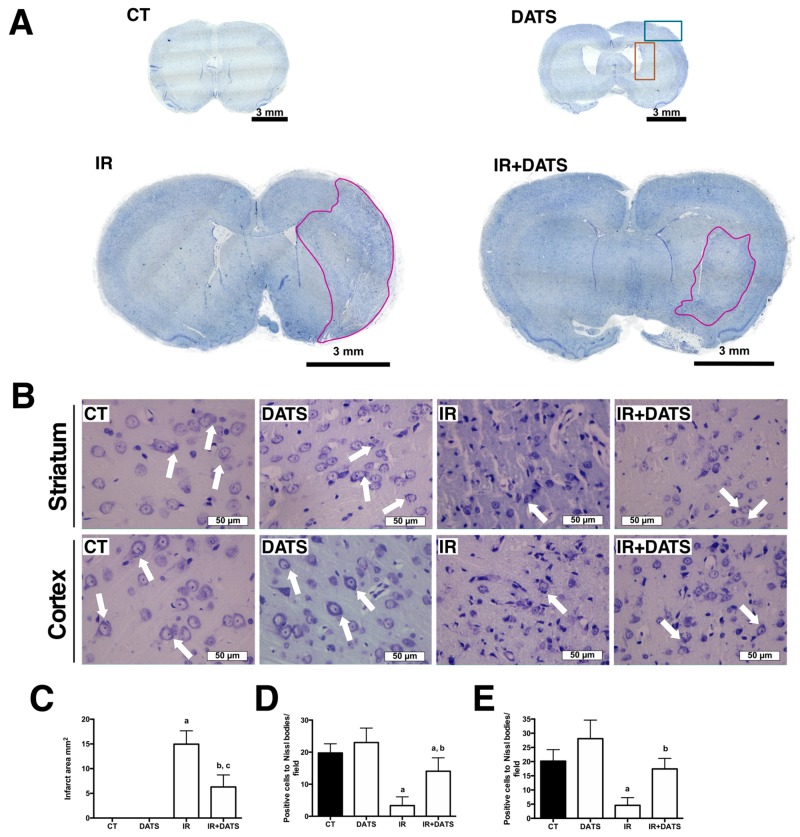
Effect of diallyl trisulfide (DATS) on the infarct area and the number of positive cells to Nissl bodies induced by ischemia/reperfusion (IR) at seven days. Animals were subjected to 1 h of ischemia and seven days of reperfusion and were administered with 4 doses of DATS (15 mg/Kg, i.p.) every 24 h, starting 5 min before the onset of reperfusion. Brains were collected and fixed 7 days after the first dose of DATS, and coronal sections (5 μm) were obtained. Nissl staining was performed and the infarct area was evaluated with the 4× objective while the number of Nissl bodies was evaluated in the striatum and cortex with the 40× objective. (**A**) Representative images of the full brains at 4× are shown. Scale bars represent 3 mm. The ocher and blue boxes represent the area of the analysis at 40×. (**B**) Representative images of the striatum and cortex at 40× are shown. Scale bars represent 50 μm. (**C**) The analysis of the infarct area in mm^2^, marked with magenta color in A, is presented. Analysis of the Nissl bodies in the images is presented as the number of positive cells to Nissl bodies/field in the striatum (**D**) and cortex (**E**). White arrows indicate positive cells to Nissl bodies. Data are expressed as mean ± SEM of 3–6 animals per group. ^a^ *p* < 0.001 and ^c^ *p* < 0.01 vs. CT group, and ^b^ *p* < 0.001 vs. IR group.

Seven days after the first dose of DATS or the onset of reperfusion, animals were anesthetized with sodium pentobarbital (200 mg/Kg, i.p.) and perfused transcardially with saline solution plus heparin (5000 U/L) followed by 4% PFA in phosphate buffered saline (PBS). Brains were removed, post-fixed in 4% PFA for 3 days, washed with distilled water, dehydrated in ethanol and xylene solutions and embedded in paraffin (McCormick Scientific, St Louis, MO, USA). Coronal sections (5 μm) were obtained in an 820 HistoSTAT microtome (American Instrument Exchange Inc. Haverhill, MA, USA) every 100 μm, covering a total distance of 300 μm. All sections were stained with hematoxylin and eosin (H&E) and visualized in a microscope (Leica, Cambdrige, UK) using the 40× objective. The analyses of the images were made from the left striatum and cortex (Figure 1A, ocher and blue boxes). The criteria to evaluate damaged tissue included pyknotic nuclei, cytoplasmic vacuolization, interstitial edema, neuronal atrophy and neuropil damage. The results are expressed as the percentage of preserved cells per field.

### 2.6. Nissl Staining

To analyze the damage produced by the IR, brain sections (5 µm) were deparaffinized in xylene, hydrated in decreasing-concentration ethanol solutions and distilled water, and stained with 0.1% cresyl violet for 15 min. Sections were rinsed with distilled water, dehydrated with absolute ethanol and xylene and mounted with DPX mounting medium. Sections were visualized in a Nikon E 200 microscope (Nikon, Melville, NY, USA) using the 4× and 40× objectives. Infarct area was evaluated with the 4× objective using the Image-Pro Insight software (Media Cynernetics, Rockville, MD, USA) and the results are expressed as infarct area (mm^2^). The number of positive cells to Nissl bodies was quantified in the left striatum and cortex (Figure 1A, ocher and blue boxes) using the 40× objective. Results are expressed as positive cells to Nissl bodies/field. A positive cell to Nissl bodies is defined as a cell that contains a large granular body of rough endoplasmic reticulum with rosettes of free ribosomes. 

### 2.7. Behavioral Testing

The evaluation of the neurological deficit was performed 10 min before the onset of reperfusion, as a cerebral ischemia indicator produced by middle cerebral artery occlusion and 30 min before the sacrifice of animals. The tests performed were: spontaneous mobility (during 10 s), contralateral turns (over a period of 30 s), forelimb flexion, grasping reflex and horizontal bar test. In the spontaneous mobility test, the rats were placed in a flat surface of 1 m^2^ and were observed for movements in a period less than 10 s. Damaged rats showed no movement during this time-period. In the contralateral turns, the rats were held from the tail and lifted up with a 45° inclination, with the forelimbs touching the flat surface, and the contralateral turns to the lesion were observed in a period of 30 s. Damaged rats showed at least 5 contralateral turns in this period of time. The forelimb flexion consisted in lifting up the rats from the tail and observing the flexion of their forelimbs. Damaged rats bent the contralateral forelimb to the lesion. The grasping reflex consisted in lifting up the rats from the tail and getting them close to a cable. Damaged rats were unable to hold the cable with both forelimbs. Finally, in the horizontal bar test, rats were suspended in a horizontal bar and the time that the rats keep holding to the horizontal bar was recorded. Damaged rats were unable to hold on for more than 5 s. In each test, a 0 value was assigned if the animal had a normal behavioral, whereas if the animal had an impairment, the assigned value was 1. The sum of scores obtained from individual tests was used to establish the degree of the neurological deficit [15]. Animals that obtained a score lower than 2 in the behavioral test 10 min before reperfusion, were excluded from the study. The results are expressed as the neurological score.

### 2.8. Nuclear Extract

The left cortex was homogenized with 500 μL of HB buffer (20 mM HEPES pH 7.4, 1 mM EDTA, 1 mM DTT, 1 mM PMSF, 1 μg/mL pepstatin A, 1 μg/mL aprotinin, 1 μg/mL leupeptin and 1 × phosphatases cocktail inhibitor) plus 0.5% Nonidet^®^ P40, and samples were incubated on ice for 15 min. Homogenates were centrifuged at 850× *g* for 10 min at 4 °C and the pellets were resuspended in 500 μL of HB buffer, incubated on ice for 10 min, followed by the addition of 15 μL of 10% Nonidet^®^ P40 and incubated for 5 min. Samples were centrifuged at 14,000× *g* for 2 min at 4 °C and the pellets were resuspended in 75 μL of complete lysis buffer (TransAM, Nrf2), vortexed 10 s and incubated on ice for 30 min with orbital agitation at 150 rpm. Samples were vortexed for 30 s, centrifuged at 14,000× *g* for 10 min at 4 °C and the supernatant (nuclear fraction) were collected and stored at −70 °C until use. 

### 2.9. Nrf2 Activation

The active Nrf2 bound to ARE sequences (Nrf2 activation) was determined by enzyme-linked immunosorbent assay (ELISA), according to the manufacturer’s instructions (TransAM Nrf2 Active Motif) using the nuclear fractions. The results are expressed as the optical density at 450 nm/μg of protein. 

### 2.10. Immunohistochemistry 

Brain sections (5 µm) were deparaffinized in xylene and hydrated in decreasing ethanol solutions and distilled water. Sections were permeabilized with PBS plus 0.2% Triton^®^ × 100 for 1 h, boiled in 10 mM sodium citrate buffer pH 6.0 plus 0.2% Triton^®^× 100 for 1 h and cooled at room temperature for 1 h. The endogenous peroxidase activity was inactivated with 1% H_2_O_2_ for 15 min. Sections were blocked with 2.5% BSA for 1 h at room temperature and incubated with primary antibody against MPP-9 (1:50) and MDA (1:250) for 24 h and SOD1 (1:100), SOD2 (1:250) and GST (1:50) for 48 h at room temperature. Sections were washed 3 times with PBS and incubated with Universal L Kit SAB-System HRP, according to manufacturer’s instructions. Finally, sections were incubated with 3′3-diaminobenzidine and counterstained with hematoxylin. Sections were visualized in a Nikon E 200 microscope (Nikon, Melville, NY, USA) using the 40× objective and 3 microphotographs were taken from 3 different zones of the left striatum and cortex (Figure 1A, ocher and blue boxes). Images were analyzed using the MIPAR 3.0 software (MIPAR Software LCC, Worthington, OH, USA). The positive area in the cells for each protein was measured and the results are expressed as the area of positive mark (μm^2^)/cell.

### 2.11. Preparation of Total Homogenates

Striatum and cortex were homogenized in cold lysis buffer (10 mM Tris HCl pH 7.6, 15 mM NaCl, 0.25 mM sucrose, 1 μg/mL aprotinin, 1 μg/mL leupeptin, 1 μg/mL pepstatin A, 1 mM PMSF and 1% Triton^®^× 100) and centrifuged at 10,621× *g* for 20 min at 4 °C. The supernatants were collected and stored at −70 °C until use. 

### 2.12. CAT Activity

Twenty-five microliters of samples were added to 725 µL of reaction mix (6 mM KH_2_PO_4_, 4 mM Na_2_HPO_4_ pH 7.0 plus 30 mM H_2_O_2_), and the absorbance was measured immediately at 240 nm every 15 s for 3 min. The activity was calculated in the time period when the reaction was lineal. A blank reaction with distilled water replacing homogenates was subtracted from each assay. The activity was calculated from the slope of the lines following a first-order kinetic. CAT activity is expressed as *k*/mg of protein.

### 2.13. GPx Activity

One hundred microliters of samples were added to 800 µL of reaction mix (30 mM KH_2_PO_4_, 20 mM Na_2_HPO_4_ pH 7.0, 1 mM EDTA, 1 mM NaN_3_, 0.2 mM NADPH, 1 mM GSH and 1 U/mL GR) and incubated for 5 min at room temperature. The reaction started by the addition of 100 µL of 0.25 mM H_2_O_2_ solution. Absorbance at 340 nm was recorded every 30 s for 3 min. In parallel, the absorbance of an unspecific tube (100 µL of distilled water, 800 µL of reaction mix and 100 µL of 0.25 mM H_2_O_2_ solution) was measured, and the value was subtracted from each assay. The activity was calculated from the slope of these lines (µmoles of NADPH oxidized per min) using the molar extinction coefficient of NADPH (6.22 × 10^3^ M^−1^ cm^−1^). GPx activity is expressed as U/mg of protein.

### 2.14. Statistical Analysis 

Data were expressed as mean ± standard error of the mean (SEM). Data were analyzed by one-way analysis of variance (ANOVA) and post hoc Tukey’s test. All data were analyzed using the software Graph Pad 5.0 (San Diego, CA, USA). Values of *p* < 0.05 were considered to be statistically significant.

## 3. Results

### 3.1. DATS Decreased the Infarct Area and Protected against Brain Damage and Motor Behavioral Impairment Induced by IR in Both Striatum and Cortex 

To determine whether DATS treatment protected against the damage induced by IR, a series of evaluations were performed in the MCAO model on rats. First, the effect of IR and DATS treatment in the infarct area was evaluated. The IR insult induced an extensive infarct area (14.96 mm^2^) while the DATS treatment decreased the damage induced by the IR (6.28 mm^2^; Figure 1A,C). Moreover, the IR insult decreased the number of positive cells to Nissl bodies in the striatum (3.33, Figure 1D) and cortex (4.6, Figure 1E) compared to CT (19.75 and 20.17, respectively) and DATS (23.00 and 28.10, respectively) groups. The administration of DATS (IR + DATS) prevented extensive tissue damage in the striatum (14.05) and cortex (17.44) that was evidenced by the preservation of Nissl bodies (Figure 1B–D).

**Figure 2:**
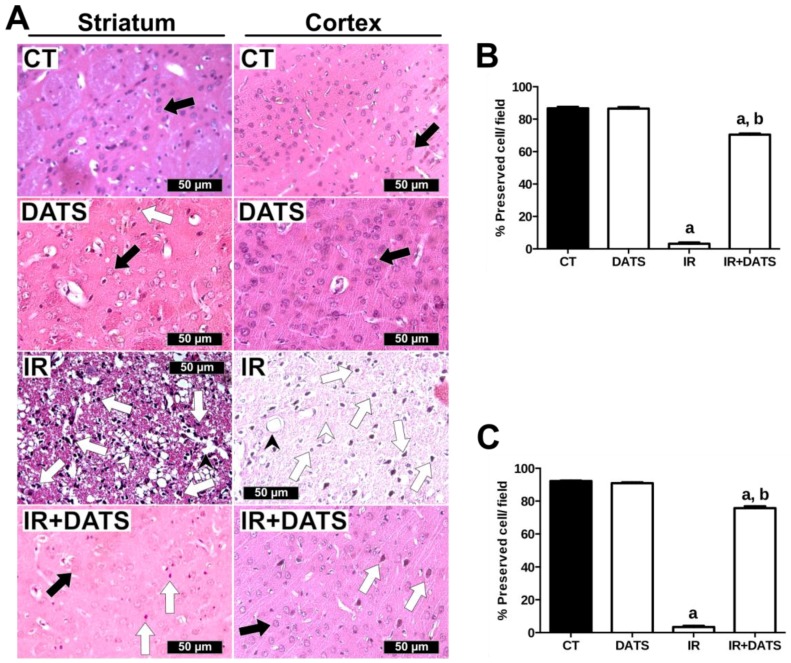
Effect of diallyl trisulfide (DATS) on brain damage induced by ischemia/reperfusion (IR) at seven days. Animals were subjected to 1 h of ischemia and seven days of reperfusion and were administered with four doses of DATS (15 mg/Kg, i.p.) every 24 h, starting 5 min before the onset of reperfusion. Brains were collected and fixed seven days after the first dose of DATS, and coronal sections (5 μm) were obtained. Hematoxylin and eosin staining were performed and 5 microphotographs per rat of the striatum and cortex were taken at 40×. (**A**) Representative images of the striatum and cortex are shown. Analysis of all images is presented as the percent of preserved cells/field in the striatum (**B**) and cortex (**C**). Black arrows indicate preserved cells, white arrows indicate pyknotic cells, black arrowheads indicate vasogenic edema and white arrowheads indicate neuropil damage. Scale bars represent 50 μm. Data are expressed as mean ± SEM of 3–6 animals per group. ^a^ *p* < 0.001 vs. CT group and ^b^ *p* < 0.001 vs. IR group.

Brain damage was also evaluated with hematoxylin and eosin staining. In contrast to the well-preserved cells and neuropils of the striatal and cortex tissues from the CT and DATS groups, the IR insult induced cellular and tissue damage such as pyknotic nuclei, vasogenic edema and neuropil damage in the striatum and cortex (Figure 2A). We also observed that the neuropil damage was more prominent in the striatum than in the cortex (Figure 2A), nevertheless the percent of preserved cells in both tissues, after IR insult, was similar in the striatum (3.19%, Figure 2B) and cortex (3.37%, Figure 2C). On the other hand, the treatment with DATS in animals subjected to IR (IR + DATS) decreased the damage induced by the IR. We observed an increase in the number of preserved cells in the striatum (70.45%, Figure 2B) and cortex (75.68%, Figure 2C). Additionally, a decrease in the number of pyknotic nuclei and vasogenic edema; and an increase neuropil integrity, compared to the IR group, were observed. The treatment with DATS alone did not change the percent of preserved cells in the striatum and cortex compared to the CT group (Figure 2).

**Figure 3:**
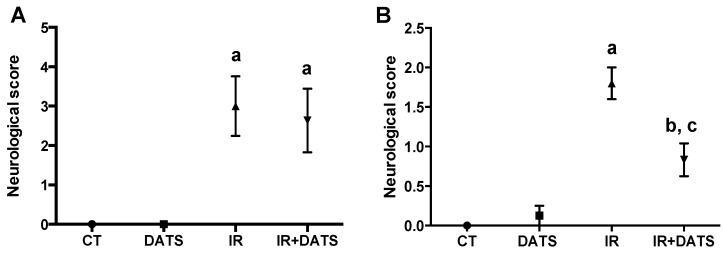
Effect of diallyl trisulfide (DATS) on the motor behavioral impairment induced by ischemia 10 min before reperfusion and ischemia/reperfusion (IR) at seven days. Animals were subjected to 1 h of ischemia and seven days of reperfusion and were administered with four doses of DATS (15 mg/Kg, i.p.) every 24 h, starting 5 min before the onset of reperfusion. The motor behavior was evaluated (**A**) 10 min before reperfusion and (**B**) 30 min before sacrifice (seven days). Data are expressed as mean ± SEM of 6 animals per group. ^a^ *p* < 0.001 and ^b^ *p* < 0.01 vs. CT group and ^c^ *p* < 0.01 vs. IR group.

Moreover, the ischemic insult induced an increase in the motor behavioral impairment before the onset of reperfusion (3.0 and 2.64, IR and IR + DATS groups; Figure 3A), whereas, seven days after reperfusion, the motor behavioral impairment remained increased (1.8, IR group; Figure 3B) compared to the CT group. The treatment with DATS (IR + DATS) prevented this motor impairment (0.83, IR + DATS group; Figure 3B) at seven days and the treatment with DATS alone (0.12, DATS group; Figure 3B) did not induce motor behavioral impairment compared to the CT group (0.0, CT group, Figure 3B).

### 3.2. DATS Prevented the Increase in the MMP-9 Levels in the Striatum and Cortex

The blood brain barrier (BBB) disruption is a common event during the IR insult as a consequence of the increased oxidative stress and the expression of MMP-9. There are reports indicating that the increase in MMP-9 induces the disruption of the BBB [30], and since we observed at a histological level, an increase in the vasogenic edema, we hypothesized that the IR insult could increase the BBB disruption through the increment in MMP-9 levels. For that reason, we evaluated the effect of DATS and the IR insult on the MMP-9 levels as an indirect marker of BBB disruption.

**Figure 4:**
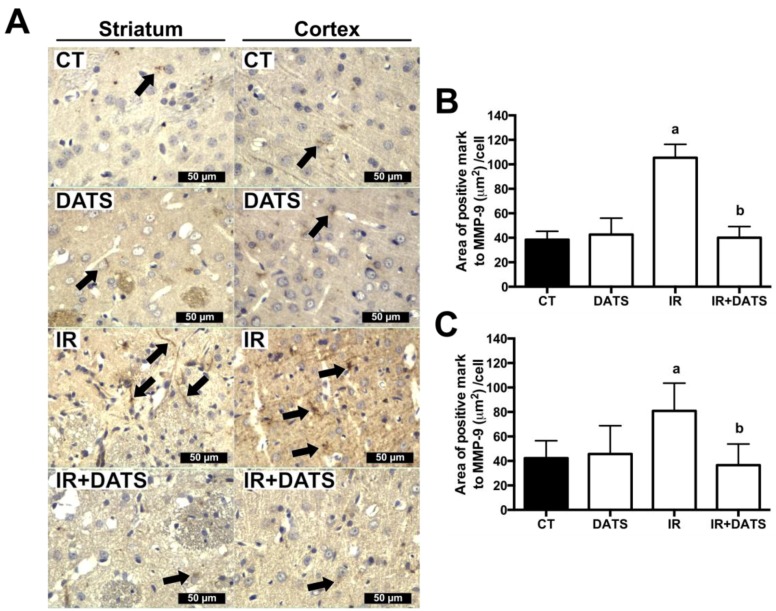
Effect of diallyl trisulfide (DATS) on the matrix metalloproteinase-9 (MMP-9) expression induced by ischemia/reperfusion (IR) at seven days. Animals were subjected to 1 h of ischemia and seven days of reperfusion and were administered with 4 doses of DATS (15 mg/Kg, i.p.) every 24 h, starting 5 min before the onset of reperfusion. Brains were collected and fixed seven days after the first dose of DATS, and coronal sections (5 μm) were obtained. Immunohistochemistry was performed to identify the expression of MMP-9 in the striatum and cortex. (**A**) Representative images of immunohistochemistry at 40× are shown. Analysis of all images is presented as the area of positive mark to MMP-9 (µm^2^)/cell in the striatum (**B**) and cortex (**C**). Black arrows indicate cells positive to MMP-9. Scale bars represent 50 μm. Data are expressed as mean ± SEM of 3–6 animals per group. ^a^ *p* < 0.001 vs. CT and DATS groups and ^b^ *p* < 0.001 vs. IR group.

The IR insult increased the levels of MMP-9 in the striatum (105.37 μm^2^/cell) and cortex (80.86 μm^2^/cell) compared with the CT (38.41 μm^2^/cell and 42.25 μm^2^/cell, respectively) and DATS groups (42.63 μm^2^/cell and 45.64 μm^2^/cell, respectively, as seen in Figure 4). The treatment with DATS in animals subjected to IR (IR + DATS) prevented the increase in MPP-9 in the striatum (40.07 μm^2^/cell) and cortex (36.52 μm^2^/cell, Figure 4).

### 3.3. DATS Decreased Oxidative Stress in the Striatum and Cortex

The oxidative stress plays an important role in the damage induced by IR [5,6,7] and since we observed that DATS treatment protected against brain damage and motor behavioral impairment induced by the IR insult and there is evidence indicating the antioxidant role of DATS [19,22,23,24], we hypothesized that DATS could decrease the oxidative stress induced by the IR insult.

**Figure 5:**
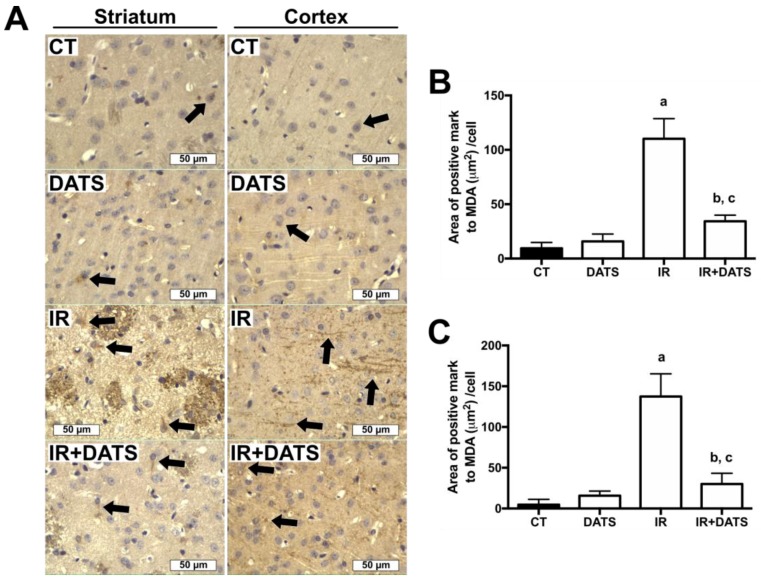
Effect of diallyl trisulfide (DATS) on the malondialdehyde (MDA) levels induced by ischemia/reperfusion (IR) at seven days. Animals were subjected to 1 h of ischemia and seven days of reperfusion and were administered with four doses of DATS (15 mg/kg, i.p.) every 24 h, starting 5 min before the onset of reperfusion. Brains were collected and fixed seven days after the first dose of DATS, and coronal sections (5 μm) were obtained. Immunohistochemistry was performed to identify the levels of MDA in the striatum and cortex. (**A**) Representative images of immunohistochemistry at 40× are shown. Analysis of all images is presented as the area of positive mark to MDA (µm^2^)/cell in the striatum (**B**) and cortex (**C**). Black arrows indicate MDA-positive cells. Scale bars represent 50 μm. Data are expressed as mean ± SEM of 3–6 animals per group. ^a^ *p* < 0.001 vs. CT group, ^b^ *p* < 0.001 vs. CT group and ^c^ *p* < 0.001 vs. IR group.

The IR insult increased the levels of MDA, a marker of lipid oxidation, in the striatum (110.15 μm^2^/cell) and cortex (137.33 μm^2^/cell) compared with the CT (9.42 μm^2^/cell and 4.75 μm^2^/cell, respectively) and DATS groups (15.83 μm^2^/cell and 15.71 μm^2^/cell, respectively, Figure 5). The treatment with DATS in animals subjected to IR (IR + DATS) decreased the levels of MDA in the striatum (32.27 μm^2^/cell) and cortex (30.08 μm^2^/cell, Figure 5), indicating that the treatment with DATS decreased the oxidative stress induced by the IR insult.

### 3.4. DATS Increased Nrf2 Activation in Cerebral Cortex

Since DATS treatment decreased brain damage, the motor behavioral impairment and the oxidative stress induced by IR and there are reports indicating the ability of DATS to induce Nrf2 activation [27], we hypothesized that the protection of DATS against IR damage in brain tissue could be associated with its ability to induce Nrf2 activation.

**Figure 6:**
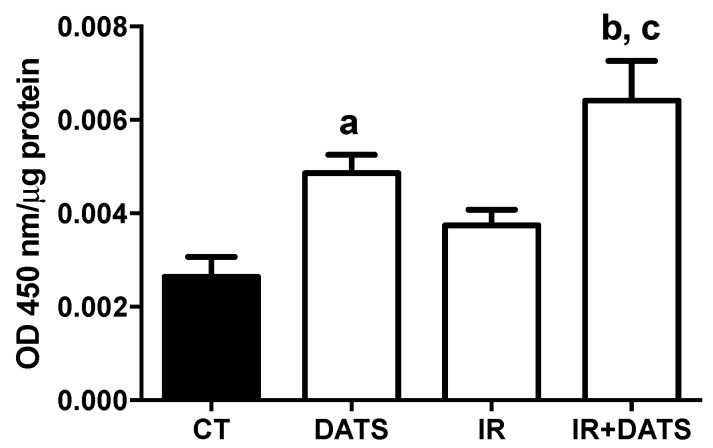
Effect of diallyl trisulfide (DATS) on the nuclear factor erythroid 2 related factor 2 (Nrf2) activation induced by ischemia/reperfusion (IR) at seven days. Animals were subjected to 1 h of ischemia and seven days of reperfusion and were administered with 4 doses of DATS (15 mg/kg, i.p.) every 24 h, starting 5 min before the onset of reperfusion. Nuclear fractions obtained from the cortex were used to evaluate the active Nrf2 binding to the ARE sequence in the ELISA plate (Nrf2 activation). Data are expressed as mean ± SEM of 6 animals per group. ^a^ *p* < 0.05 vs. CT group, ^b^ *p* < 0.001 vs. CT group and ^c^ *p* < 0.01 vs. IR group.

The IR insult did not increase the Nrf2 activation compared to the CT group. Conversely, the treatment with DATS in animals subjected to IR (IR + DATS) increased 1.42-fold the transcriptional activation of Nrf2 compared to the CT group and 0.72-fold compared to IR group. Moreover, the treatment alone with DATS increased 0.88-fold the transcriptional activation of Nrf2 compared to the CT group (Figure 6).

### 3.5. DATS Increased the Levels of SOD1 and SOD2 in Striatum and Cortex 

Nrf2 is a transcription factor capable to induce the expression of proteins related to the antioxidant response such as SOD [31] and, because we observed an increase in the Nrf2 transcriptional activity after DATS administration and decrease in oxidative stress, we hypothesized that DATS could increase the expression of SOD1 and SOD2, as a possible mechanism of protection against IR injury. To test this, we evaluated SOD1 and SOD2 levels by immunohistochemistry.

**Figure 7:**
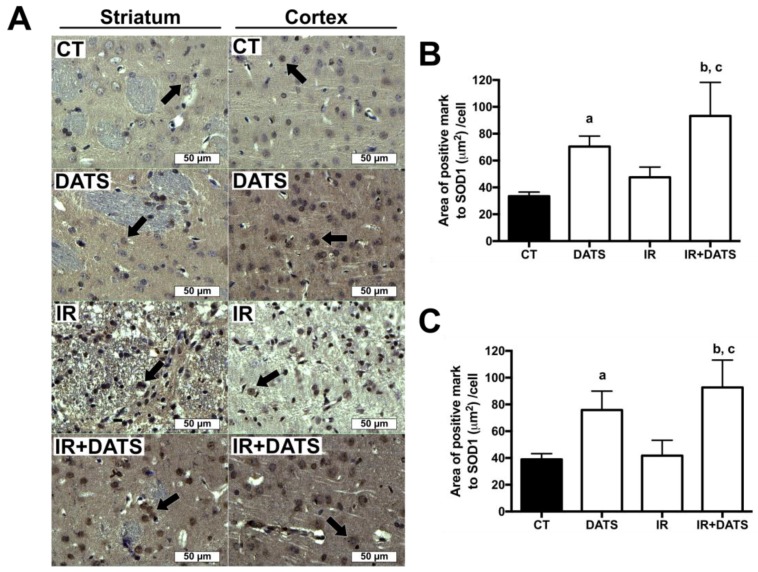
Effect of diallyl trisulfide (DATS) on the expression of superoxide dismutase 1 (SOD1) induced by ischemia/reperfusion (IR) at seven days. Animals were subjected to 1 h of ischemia and seven days of reperfusion and were administered with four doses of DATS (15 mg/Kg, i.p.) every 24 h, starting 5 min before the onset of reperfusion. Brains were collected and fixed seven days after the first dose of DATS, and coronal sections (5 μm) were obtained. Immunohistochemistry was performed to identify the expression of SOD1 in the striatum and cortex. (**A**) Representative images of immunohistochemistry at 40× are shown. Analysis of all images is presented as the area of positive mark to SOD1 (µm^2^)/cell in the striatum (**B**) and cortex (**C**). Black arrows indicate SOD1-positive cells. Scale bars represent 50 μm. Data are expressed as mean ± SEM of 3–6 animals per group. ^a^ *p* < 0.001 vs. CT group, ^b^ *p* < 0.001 vs. CT group and ^c^ *p* < 0.001 vs. IR group.

The IR insult tend to increase the expression of SOD1 (47.55 μm^2^/cell) in damaged cells of the striatum compared to the CT group (33.45 μm^2^/cell). The treatment with DATS in animals subjected to IR (IR + DATS) increased the expression of SOD1 in the striatum (93.26 μm^2^/cell) and cortex (92.73 μm^2^/cell) compared to all the other groups. The treatment with DATS alone increased the expression of SOD1 in the striatum (70.47 μm^2^/cell) and cortex (75.88 μm^2^/cell) compared to the CT group (33.46 μm^2^/cell and 38.95 μm^2^/cell, respectively); nevertheless, we observed higher expression of SOD1 in the cortex compared to the striatum. Unexpectedly, the expression of SOD1 in DATS, IR and IR + DATS groups was observed mainly in the nuclear sub-cellular compartment; while in the CT group it was perinuclear (Figure 7).

**Figure 8:**
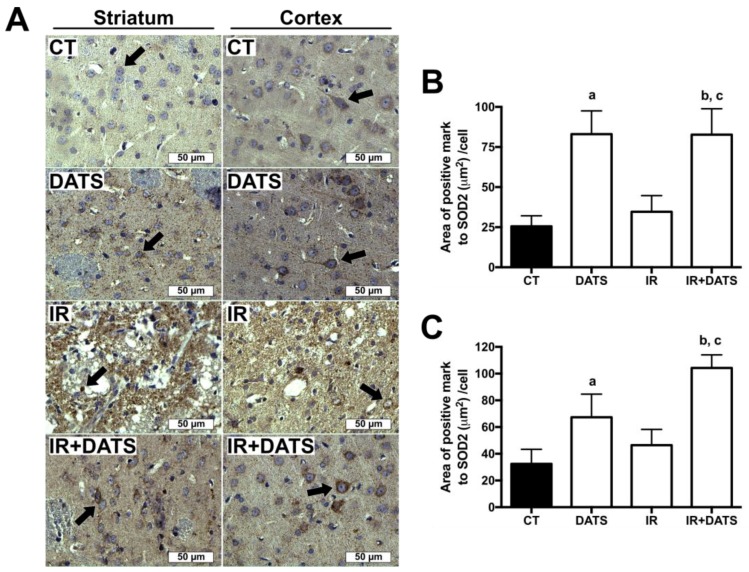
Effect of diallyl trisulfide (DATS) on the superoxide dismutase 2 (SOD2) expression induced by ischemia/reperfusion (IR) at seven days. Animals were subjected to 1 h of ischemia and seven days of reperfusion and were administered with four doses of DATS (15 mg/kg, i.p.) every 24 h, starting 5 min before the onset of reperfusion. Brains were collected and fixed at seven days after the first dose of DATS, and coronal sections (5 μm) were obtained. Immunohistochemistry was performed to identify the expression of SOD2 in the striatum and cortex. (**A**) Representative images of immunohistochemistry at 40× are shown. Analysis of all images is presented as the area of positive mark to SOD2 (µm^2^)/cell in the striatum (**B**) and cortex (**C**). Black arrows indicate SOD2-positive cells. Scale bars represent 50 μm. Data are expressed as mean ± SEM of 3–6 animals per group. ^a^ *p* < 0.001 vs. CT group, ^b^ *p* < 0.001 vs. CT group and ^c^ *p* < 0.001 vs. IR group.

The IR insult tends to increase SOD2 expression in damaged cells of the striatum (34.63 μm^2^/cell) and cortex (46.41 μm^2^/cell) compared to CT group (25.55 μm^2^/cell and 32.43 μm^2^/cell, respectively). The treatment with DATS in animals subjected to IR (IR + DATS) increased the expression of SOD2 in the striatum (82.73 μm^2^/cell) and cortex (104.28 μm^2^/cell) compared to all groups. The treatment alone with DATS increased SOD2 levels in the striatum (83.08 μm^2^/cell) and cortex (67.33 μm^2^/cell) compared to CT group. Finally, we observed that SOD2 expression is higher in the cortex than the striatum in CT, IR and IR + DATS groups (Figure 8).

### 3.6. DATS Increased the GST Enzyme Levels in Both Striatum and Cortex 

The GST enzyme has an ARE sequence in their promoter region, as well as SOD1 and SOD2, and there are reports indicating that Nrf2 activation induces GST expression [32], for this reason, we investigated whether DATS treatment is able to increase the expression of GST in the striatum and cortex. 

**Figure 9:**
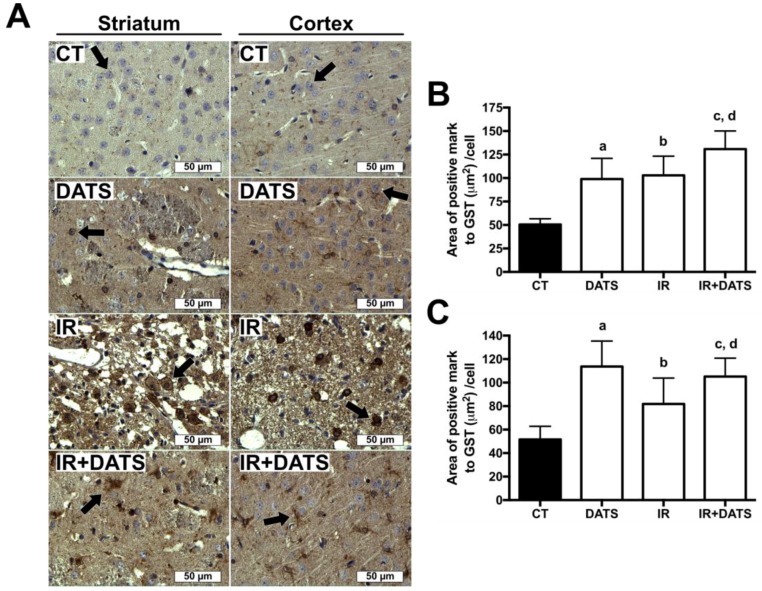
Effect of diallyl trisulfide (DATS) on the glutathione S-transferase (GST) induced by ischemia/reperfusion (IR) at seven days. Animals were subjected to 1 h of ischemia and seven days of reperfusion and were administered with four doses of DATS (15 mg/Kg, i.p.) every 24 h, starting 5 min before the onset of reperfusion. Brains were collected and fixed seven days after the first dose of DATS, and coronal sections (5 μm) were obtained. Immunohistochemistry was performed to identify the expression of GST in the striatum and cortex. (**A**) Representative images of immunohistochemistry at 40× are shown. Analysis of all images is presented as the area of positive mark to GST (µm^2^)/cell in the striatum (**B**) and cortex (**C**). Black arrows indicate GST-positive cells. Scale bars represent 50 μm. Data are expressed as mean ± SEM of 3–6 animals per group. ^a^ *p* < 0.001 vs. CT group, ^b^ *p* < 0.001 vs. IR group, ^c^ *p* < 0.001 vs. CT group and ^d^ *p* < 0.05 vs. IR group.

The IR insult increased GST expression in damaged cells of the striatum (102.85 μm^2^/cell) and cortex (81.86 μm^2^/cell) compared to CT group (50.47 μm^2^/cell and 51.61 μm^2^/cell). The administration of DATS in animals subjected to IR (IR + DATS), induced the major expression of GST in the striatum (130.85 μm^2^/cell) and cortex (105.12 μm^2^/cell) compared with all groups, except on DATS group in cortex. The treatment alone with DATS increased the expression of GST in both regions (98.99 μm^2^/cell and 113.67 μm^2^/cell, respectively, Figure 9).

### 3.7. DATS Did not Increase the Activity of CAT and GPx

We observed that the protective effect of DATS against the IR insult occurred through Nrf2 activation and there are reports indicating that CAT and GPx are two enzymes regulated by Nrf2, for this reason, we tested whether the DATS treatment increased the activity of these enzymes.

The IR insult increased the enzyme activity of CAT in the striatum and GPx in the cortex compared to the CT group, whereas the treatment with DATS (IR + DATS) increased the enzyme activity of CAT and GPx in the striatum and cortex, respectively, in a similar way to that observed in the IR group. The treatment with DATS alone did not increase the enzyme activity of CAT and GPx in the striatum and cortex compared to the CT group (Table 1).

## 4. Discussion

Despite the fact that cerebral ischemia is the leading cause of adult disability and the second cause of death worldwide, the number of therapies against stroke is very limited [3,33]. The U.S. Food and Drug Administration (FDA) has only approved the use of the tissular plasminogen activator (t-PA) for the treatment of acute ischemic stroke up to 4.5 h after symptoms onset; however, not all patients are candidates for this therapy. The treatment with t-PA is a thrombolytic therapy focused on restoring brain blood flow. Nonetheless, there are no treatments against the biochemical and molecular events triggered during IR that are important for the tissue damage, such as ROS production Reviewed in [34]. ROS are important mediators in cerebral tissue damage because of their ability to oxidize proteins, lipids and nucleic acids [7]. Their production takes place during the IR injury, mainly in the reperfusion, and their highest production occurs after the first 20 min of reperfusion and extends until 3 h [6]. Considering the role of ROS in tissue damage in the stroke, the evaluation of antioxidant compounds against IR injury is an attractive alternative for the treatment of cerebral ischemia.

DATS is an organosulfur compound with direct and indirect antioxidant properties [22,27]. It is also a hydrophobic molecule with predicted ability to cross the blood brain barrier [22], making it an attractive molecule for the treatment of neurological diseases. There is evidence indicating its protective role against oxidative stress induced in myocardial-ischemia [26,35] and in an oxygen-glucose deprivation model in neuronal cells [36]; however, in an in vivo model of cerebral ischemia, this compound had not been evaluated until now. 

We hypothesized that the administration of DATS could prevent the cerebral damage induced by ROS in the MCAO model. We observed a prominent infarct area in animals subjected to IR insult, as well as a decrease in the number of cells positive to Nissl bodies. At the histological level, the IR insult decreased the number of preserved cells in the striatum and cortex; however, the treatment with DATS decreased the infarct area and prevented the cellular damage induced by IR. Moreover, at the cellular level, DATS prevented the damage induced by IR in the striatum and cortex, in a similar way, even at the neuropil level. We consider that the protective role of DATS in the brain tissue is associated with its antioxidant properties, since we observed a decrease in the MDA levels induced by the IR insult. DATS is capable to act as direct and indirect antioxidant [22,27]. We considered that the first dose of DATS (5 min before reperfusion) could be involved in ROS removal, generated during the first hour after reperfusion, due to its ability to scavenge ROS [22]; then again, the following three doses of DATS could be acting as indirect antioxidants, inducing Nrf2 activation and subsequent expression of antioxidant and phase 2 proteins, which maintain the redox balance in cells and neutralize the ROS generated at later times.

The middle cerebral artery supplies blood flow to the striatum, which regulates the beginning and the end of movements, as well as to the superior frontal and parietal cortex, which are involved in the voluntary control of movements [37]. The disruption of the cerebral blood flow, as a consequence of the occlusion of the middle cerebral artery induced tissular damage in the striatum and cortex, resulting in motor function impairment and long-term disability [38]. Considering the effect of the IR in motor function, we evaluated the protective effect of DATS at this level. We observed in animals subjected to IR and administered with DATS, an improvement in the motor function compared with animals of the IR group, correlating with the decrease in the infarct area and morphological alterations, and the increase in the number of cells positive to Nissl bodies; which suggests that the protective effect of DATS could be associated with the amelioration of brain damage and oxidative stress. Our results are consistent with other reports, which observed that the antioxidant compounds treatment improve of motor function and ameliorate brain damage induced in cerebral ischemia models [17]. Specifically, there is evidence indicating that garlic oil with high concentration of DATS, protected the brain tissue against the IR insult by reducing the infarct volume and improving the behavioral impairment in mice [39].

The BBB disruption due to ROS production and matrix metalloproteinases (as MMP-9) is an important mechanism of brain damage in stroke [30]. The treatment with DATS in animals subjected to IR decreased the levels of MMP-9 and oxidative stress, which could be associated with the decreases in the BBB disruption and brain tissue damage, since we observed at the histological level, a decrease in vasogenic edema.

As mentioned above, the protective effect of DATS at the histological and motor function levels could be associated with its direct and indirect antioxidant properties since we observed a decrease in the oxidative stress; however, we focused on evaluating its indirect antioxidant properties. Indirect antioxidants induce Nrf2 activation and increase the endogenous antioxidant defense, triggering a long-term protection against oxidative stress, compared with direct antioxidants which react with ROS and are consumed, generating a short-term protection [40].

There are reports indicating that DATS induced Nrf2 accumulation and activation in HepG2 [28] and in NSC34 cells [41] and induced the expression of NQO1 in lumbar spinal cord explants [42]. Moreover, DATS induced the expression of Nrf2 and antioxidant enzymes in renal tissue [43], and protected B35 neuronal cells through Nrf2 activation after oxygen and glucose deprivation [36]. This evidence indicates that DATS induced the activation of the Nrf2 pathway. For these reasons, we evaluated the effect of DATS on Nrf2 activation as a possible mechanism of protection against the IR insult. Nrf2 activation was observed in healthy rats administered with DATS, while the administration of DATS in animals subjected to IR (IR + DATS) also induced Nrf2 activation. However, in the IR + DATS group, the Nrf2 activation was greater, suggesting a synergistic effect of the IR insult and DATS treatment on Nrf2 activation. These results support our hypothesis that the protective effect of DATS observed at the histological level and in motor function occurred through Nrf2 activation.

The protein Keap1 regulates Nrf2 activation. In homeostatic redox conditions, Keap1 interacts with Nrf2 in the cytoplasm [10]. This interaction induces the Nrf2 ubiquitination and its subsequent degradation by the 26S proteasome, preventing the nuclear translocation of Nrf2 and its binding to the ARE sequence (Nrf2 activation) [11]. The Keap1-Nrf2 interaction is carried out through the Kelch domain in Keap1 and the ETGE and DLG motifs in Nrf2 [12]. However, there are some cysteine residues in Keap1, such as C151, C273 and C288, important in Nrf2 repression [44]. These residues are reactive cysteines involved in Nrf2 activation, since they are targeted by different Nrf2 inducers [13]. Kim et al. [27] found that Nrf2 activation induced by DATS in AGS cells occurred by the direct interaction between DATS with the C273 in Keap1 and it is probable that the Nrf2 activation observed in this work could be associated with the interaction of DATS with C273 in Keap1.

Nrf2 is a transcription factor capable of regulating the expression of around 1055 genes containing ARE sequences, which include phase 2 enzymes, antioxidant enzymes and proteins involved in cellular development, metabolism, immune system and cellular signaling [45]. However, the main interest of the Nrf2 transcription factor is its ability to increase the endogenous antioxidant defense, such as SOD, GST, GPx, CAT, and others [30]. According to our results about Nrf2 activation after treatment with DATS, we evaluated the levels and activity of some enzymes regulated by Nrf2, to determine whether Nrf2 activation induced an increase in the endogenous antioxidant defense, triggering a long-term protection against oxidative stress. Our results show an increase in the levels of SOD1 and SOD2 in the striatum and cortex of animals treated with DATS and subjected to IR (IR + DATS), whereas in animals treated with DATS, the levels of these enzymes was also increased, however the expression of these enzymes was lower than in the IR + DATS group; correlating with the Nrf2 activation. The increases in SOD1 and SOD2 expression as a consequence of DATS treatment were observed previously in H9c2 cells and in the renal tissue of rats [43,46]. Unexpectedly, we observed a nuclear localization of SOD1 in DATS, IR and IR + DATS groups. This nuclear translocation of SOD1 could be associated with its ability to neutralize ROS, preventing DNA oxidation. According with our results, there is evidence indicating that SOD1 is translocated to the nucleus in primary cultured human lens epithelial cells (HLEC) after treatment with 17β-estradiol [47]. The nuclear translocation of SOD1 requires its phosphorylation by the ataxia-telangiectasia-mutated/checkpoint kinase 2 (ATM/Chk2) [48], two kinases activated by oxidative stress [49] and DATS [50]. There are reports indicating that the physiological role of nuclear SOD1 is associated with the decrease in the DNA damage induced by ROS in yeast [51] and in neuroblastoma SH-SY5Y cells [48]. Another possibility of the role of SOD1 in the nucleus is its ability to function as a transcription factor. In yeast, SOD1 was able to induce the expression of genes related to oxidative stress defense, replication stress, DNA damage response, general stress response and Cu/Fe homeostasis [51]; however, this alternative needs to be evaluated in mammals.

We observed that the expression of GST increased in animals treated with DATS in both, the striatum and cortex; while the levels of GST in animals treated with DATS and subjected to IR (IR + DATS) were even higher. Correlating with our results, there are reports indicating that DATS was capable of inducing the expression of GST in rat clone 9-derived cells in a Nrf2 dependent manner [52].

Moreover, we also measured the activity of CAT and GPx, two antioxidant enzymes involved in H_2_O_2_ clearance Reviewed in [53]. We observed an increase in the activities of CAT and GPx in the striatum and cortex, respectively, in animals subjected to IR and in animals subjected to IR and treated with DATS (IR + DATS). However, DATS alone failed to induce an increase in CAT and GPx activities, suggesting that during this period of time, the treatment with DATS had no effect on the CAT and GPx enzymes.

SOD1, SOD2, GST, CAT and GPx have ARE sequences in their gene sequence and are regulated by the transcription factor Nrf2 and in consequence by Nrf2-activator compounds [31], such as DATS. However, we did not observe an increase in CAT and GPx with DATS treatment, suggesting that DATS could regulate in a different way the expression of different genes through the Nrf2 pathway. Supporting our results, there are reports indicating that treatment with DATS increased the activity of GST and GR in hepatic tissue while in red blood cells, it did not modify the activity of these enzymes [54]. In hepatic tissue of mice, the treatment with DATS in animals subjected to acute ethanol exposure increased the activity of GR, SOD and CAT, but it did not affect the activity of GPx [55]. Liu et al. [41] reported that DATS induced, in a Nrf2-dependent manner, the expression of HO-1 and NQO1, however the levels of glutamate-cysteine ligase catalytic subunit (GCLC) and glutathione synthetase were not affected in NSC34 cells. The expression of genes regulated by Nrf2 is a differential process. There are reports indicating that the acetylation of Nrf2 induced the expression of some genes such as NQO1, thioredoxin reductase 1 (TXNRD1) and glutamate-cysteine ligase modifier subunit (GCLM), while the expression of other genes such as HO-1 were not affected, suggesting that Nrf2 acetylation promotes the specific binding of Nrf2 to the promoter of some genes [56].

**Figure 10:**
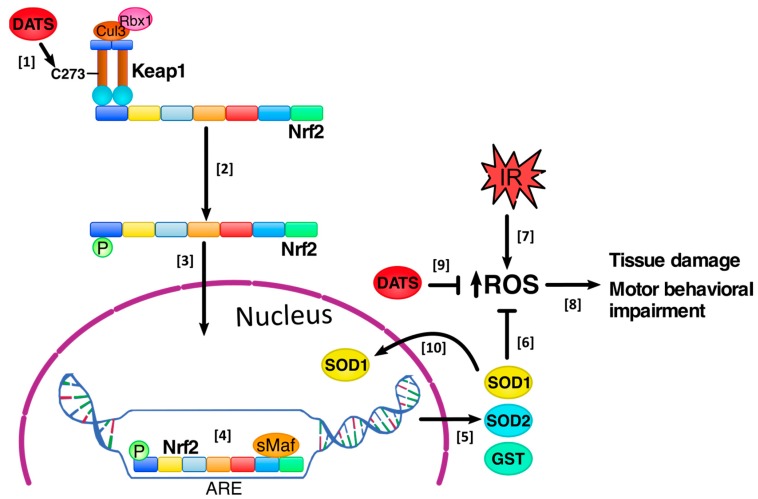
Diallyl trisulfide (DATS) protected the brain tissue against the damage induced by ischemia/reperfusion (IR). [1] DATS administration every 24 h for 4 days, starting 5 min before the onset of reperfusion, induced the release of the nuclear factor erythroid 2 related factor 2 (Nrf2) from the Kelch like ECH-associated protein 1 (Keap1), possibly through the direct interaction between DATS with the cysteine 273 (C273) in Keap1, inducing a conformational change in Keap1’s structure, [2] releasing Nrf2 and inducing its phosphorylation and [3] nuclear translocation. [4] In the nucleus, Nrf2 is dimerized with small musculoaponeurotic fibrosarcoma (sMaf) proteins and binds to the antioxidant response elements (ARE), [5] inducing the expression of antioxidant enzymes such as superoxide dismutase 1 (SOD1) and SOD2 and the phase 2 enzyme glutathione S-transferase (GST) [6]. These enzymes decrease reactive oxygen species (ROS) levels induced by the IR insult. [7] The IR induced an increase in ROS production, [8] which induce tissue damage and motor behavioral impairment; however, DATS treatment avoided tissue damage and improved motor behavioral impairment. [9] Additionally, there is a possibility that DATS reacted with the ROS generated during the IR, reducing their levels due to its direct antioxidant properties, protecting the brain tissue [10]. Finally, DATS treatment increased the total levels of SOD1 and its nuclear translocation, which could be important in preventing DNA oxidation by ROS, maintaining genomic stability. Rbx1 = RING-box protein 1, Cul3 = Cullin-3.

Based on our results, we suggest that DATS increased Nrf2 activation, inducing long-term protection against oxidative stress through the expression of SOD1, SOD2 and GST, which decrease the oxidative stress in the striatum and cortex, protecting the brain cells from the damage induced by cerebral IR, as observed in the infarct area, at the histological level and the improvement in motor behavior. There is a possibility that DATS reacted with ROS, reducing their levels due to its direct antioxidant properties. Additionally, DATS also increased SOD1 nuclear translocation, which could be important in preventing DNA oxidation by ROS, maintaining genomic stability (Figure 10).

The present study demonstrated that DATS protected the brain tissue against the damage induced by the IR insult, at the histological and motor behavioral levels, and this protection is associated with the decrease in oxidative stress through Nrf2 activation induced by DATS.

## Figures and Tables

**Table 1:** Effect of diallyl trisulfide (DATS) on the activities of catalase (CAT) and glutathione peroxidase (GPx).

Group	Striatum CAT Activity (k/mg Protein)	Cortex CAT Activity (k/mg Protein)	Cortex GPx Activity (U/mg Protein)
CT	0.003193 ± 0.000392	0.003133 ± 0.000306	0.004643 ± 0.001362
DATS	0.003388 ± 0.000236	0.001608 ± 0.000055	0.006772 ± 0.001077
IR	0.005246 ± 0.000299 ***	0.004442 ± 0.000337	0.009219 ± 0.001125 *
IR + DATS	0.005583 ± 0.000359 ***	0.004105 ± 0.000898	0.010490 ± 0.001646 **

Data are expressed as mean ± SEM of 6 animals per group. * *p* < 0.05, ** *p* < 0.01 and *** *p* < 0.001 vs. CT group.
